# Effect of PilE4 of *Francisella tularensis *subsp. holarctica (live vaccine strain) to brain endothelium

**DOI:** 10.1186/1756-3305-7-S1-P10

**Published:** 2014-04-01

**Authors:** E Bencurova, R Mucha, L Pulzova, M Bhide

**Affiliations:** 1 Laboratory of biomedical microbiology and immunology, University of veterinary medicine and pharmacy in Kosice, Kosice, Slovakia; 2 Institute of Neuroimmunology, Slovak Academy of Science, Bratislava, Slovakia

## 

*Francisella *are rare, highly infectious vector-borne bacteria, which infect more than 250 host species, including humans. *Francisella *are able to invade multiple organs in the host body, such a skin, liver, lung and central nervous system. To invade various organs, *Francisella *have to cross the cell linings like endothelial barriers. Interaction with endothelial cells is multistage process, which includes adhesion and activation of signaling events. Our previous work has shown that *Francisella tularensis *subsp. *holarctica *Live Vaccine Strain (LVS) interacts and adhere to endothelial cells through PilE4 and Intercellular adhesion molecule 1(ICAM-1) interaction. To corroborate consequences of PilE4 adhesion to endothelial cells, we incubated brain microvascular endothelial cells with recombinant PilE4 protein and assessed the regulation of several genes with qRT-PCR. Results showed that PilE4 upregulated the expression of adhesion molecules (ICAM-1 and PECAM-1), matrix metalloproteases (MMP 1, MMP 3 and MMP 9) and molecules that are involved in pathogen recognition (TLR-6, MyD88, IRAK-1 and TRAF-6) in brain endothelium. On the other hand, we found down regulation of interleukins and NF-kB. Inhibition of NF-kB indicates that *Francisella *might use NF-kB subversion mechanism to evade immune response. This mechanism is described in other pathogens such as *Listeria *and *Yersinia*. Previous studies have showed that matrix-metalloprotases, mainly MMP-9, is crucial to disrupt cytoskeleton of brain endothelium to increase the permeability of BBB. In summary, work reveal hidden aspects of invasion and translocation of *Francisella *across the brain endothelium, that offers new insight into the pathobiology of neuroinvasion of *Francisella*.

